# Three-Dimensional Surface Reconstruction for Specular/Diffuse Composite Surfaces

**DOI:** 10.3390/s24247942

**Published:** 2024-12-12

**Authors:** Chung-Hsuan Huang, Ssu-Chia He, Tsung-Yu Chen, Chau-Jern Cheng, Han-Yen Tu

**Affiliations:** 1 Institute of Electro-Optical Engineering, National Taiwan Normal University, Taipei 11677, Taiwan; terry.chhuang@gmail.com (C.-H.H.); jessy8538@gmail.com (S.-C.H.); cjcheng@ntnu.edu.tw (C.-J.C.); 2 Department of Electrical Engineering, Chinese Culture University, Taipei 11114, Taiwan; a0973865016@gmail.com

**Keywords:** digital holography, fringe projection, three-dimensional shape measurement

## Abstract

This paper presents an effective three-dimensional (3D) surface reconstruction technique aimed at profiling composite surfaces with both specular and diffuse reflectance. Three-dimensional measurements based on fringe projection techniques perform well on diffuse reflective surfaces; however, when the measurement targets contain both specular and diffuse components, the efficiency of fringe projection decreases. To address this issue, the proposed technique integrates digital holography into the fringe projection setup, enabling the simultaneous capture of both specular and diffuse reflected light in the same optical path for full-field surface profilometry. Experimental results demonstrate that this technique effectively detects and accurately reconstructs the 3D profiles of specular and diffuse reflectance, with fringe analysis providing the absolute phase of composite surfaces. The experiments validate the effectiveness of this technique in the 3D surface measurement of integrated circuit carrier boards with chips exhibiting composite surfaces.

## 1. Introduction

Optical three-dimensional (3D) shape measurement techniques have been widely researched and increasingly adopted in scientific and precision applications, including advanced manufacturing, industrial automation, reverse engineering, quality control, and biomedical and mechanical engineering. These techniques, known for their non-contact nature, fast measurement speeds, and high accuracy, are extensively employed to detect defects and verify product quality. In recent years, 3D shape measurement technologies have matured significantly, and alternative methods such as stereo vision, laser scanning, and structured light techniques are now widely used across industries. However, these methods often rely on uniform illumination for accurate sensing, and variations in surface reflectivity can significantly impact measurement reliability.

In 3D inspection, it is typically assumed that the object’s surface reflects light in a perfectly diffuse, non-absorbent manner. However, this assumption is problematic when measuring composite surfaces with both specular and diffuse reflections, such as those found in integrated circuit (IC) carrier boards with shiny chips. Many industrial IC chips are fabricated on silicon wafer substrates with high surface reflectivity, creating challenges for 3D profilometric techniques. IC carrier boards and chips are essential components of digital and analog circuits, and the accurate measurement of their 3D surfaces comprising both specular and diffuse reflective regions is crucial to ensuring that their design specifications are met for reliable manufacturing.

Common methods for 3D surface measurement include the stylus probe method [1] and the extended depth of field method [2]. The stylus probe method is renowned for its ability to provide highly precise surface profile measurements. However, as a contact-based scanning technique, it carries the risk of damaging the sample surface and requires considerable time to complete. Additionally, its accuracy may be compromised when measuring diffuse surfaces due to unevenness or roughness. The extended depth of field method, in contrast, utilizes bright-field microscopy to adjust the image’s focal plane and employs algorithms to extract imaging areas from different focal depths. These extracted imaging regions are then synthesized to construct the 3D surface profile of the object. Despite its utility, the extended depth of field method involves complex precision focusing and image stitching mechanisms. Another challenge lies in the difficulty of distinguishing between specular and diffuse regions using bright-field microscopy.

The existing 3D optical surface measurement techniques, digital holography (DH) [3], and structured light projection methods [4] have been extensively studied due to their advantages of non-destructive, high-speed, and full-field measurement capabilities. DH is a powerful 3D imaging technology based on wavefront reproduction. It records the wavefront information of an object through interference, creating a digital hologram. Using numerical reconstruction, the 3D surface of the object can be reconstructed and analyzed quantitatively [5]. Structured light projection techniques are widely utilized in industrial applications due to their simple setup and high robustness. Among these, the fringe projection technique stands out as one of the most promising methods for 3D surface profiling, offering fast speed, high accuracy, and non-contact measurement. In this technique, structured light patterns are projected onto the object and deformed according to its 3D shape, with the resulting fringe variations representing the object’s 3D distribution. Phase-shifting fringe projection (PFP) is employed to extract phase information corresponding to the object’s height through phase-wrapping calculations. The wrapped phase maps are then processed using phase unwrapping algorithms to convert phase information into height data [6].

However, while structured light projection techniques can deliver rapid and accurate measurements, they typically assume that the surfaces being measured exhibit diffuse reflectance. When images captured include specular reflections, this assumption can lead to significant phase errors due to the violation of uniform illumination conditions. To address the challenge of measuring shiny surfaces, numerous methods have been proposed, including polarized imaging, exposure control, and optimal viewing arrangements [7,8,9]. Nonetheless, these approaches often necessitate complex calibration or multiple reflections from various angles during measurement, complicating the acquisition of accurate phase information for specular surfaces. Comparative studies on the sensitivity and accuracy of fringe projection and fringe reflection techniques highlight their performance on partially diffuse and specular targets. In some cases, initial height information is estimated and used as input for slope calculations to facilitate measurements on specular surfaces [10]. Additionally, researchers have explored light separation strategies, employing polarization or color analysis to differentiate between reflection components [11,12]. Various methods have been developed to estimate maximum illumination chromaticity and intensity or to convert image color to the hue domain for mitigating saturation issues [13]. Techniques such as multi-exposure and the projection of specially structured light have also been implemented to address non-uniform light reflections [14,15]. While these methods have shown effectiveness for specific surface reflectance scenarios, they are often limited by conditions related to slope integration or maximum detectable surface curvature, thus hindering absolute phase measurement accuracy [16,17].

Digital holography, while applicable for specular reflective specimens, is often challenged by the presence of both specular and diffuse reflection characteristics in many practical applications. Polished or coated surfaces can alter reflectivity and complicate 3D shape measurement [18,19]. Such pre-processing techniques can modify surface properties, underscoring the need for rapid and high-quality technological advancements in 3D composite surface measurement and inspection. Consequently, more researchers are focusing on techniques that provide 3D profiles of surfaces with both specular and diffuse reflection. Some studies have attempted to combine fringe projection and phase measurement deflection methods to measure partially diffuse and specular targets. However, these systems typically fail to measure the target comprehensively in a single pass [20]. Other research combines gradient changes in reflected illumination with fringe projection height changes, employing iterative algorithms for shape reconstruction of different reflective objects. Due to the gradient integration processes in highly reflective areas, the integration of various subsystems into a cohesive system remains essential for achieving accurate 3D surface measurements, often reducing overall efficiency [21,22].

Given the above analysis, it is evident that each current optical 3D measurement method possesses unique advantages and disadvantages. Many methods struggle to simultaneously provide both accuracy and efficiency when measuring highly reflective surfaces. As a result, researchers are increasingly exploring ways to integrate these attributes. Several optical measurement techniques have been successfully combined using high-speed non-contact optical multi-sensor systems for rapid 3D shape acquisition across multiple scales [23,24]. Additionally, contact probe techniques have been utilized to enhance measurement accuracy [25,26]. However, it is crucial to recognize that each 3D measurement system operates within its own coordinate system, necessitating the integration of results from different systems into a unified field of view (FOV) to form a complete 3D model. This integration requires careful identification of measurement features and the automatic calculation of common reference coordinates for effective registration and calibration [27,28].

Many semiconductor components often have complex geometric shapes exhibiting both specular and diffuse reflections. These parts require precise detection by optical sensors and thorough inspection during manufacturing to ensure compliance with strict specifications and tolerances. Consequently, developing an effective technique to address the challenges posed by complex surface reflectivity in optical inspections is crucial. One primary challenge is managing the wide range of surface reflectance, which includes both specular and diffuse reflections. Variations in reflected light detected by imaging sensors can significantly impact the accuracy of surface shape measurements, underscoring the importance of an approach that can effectively handle such complexities.

Research indicates that an effective system for measuring 3D composite surfaces requires a full-field integration of the surface data, with sufficient overlapping feature points for reliable image stitching. In cases where structures have very similar 3D heights or shapes, this image stitching becomes challenging, often leading to unreliable and inefficient measurements. To achieve accurate and efficient 3D shape measurements, an integrated 3D measurement method has been designed. This paper proposes an effective optical configuration that combines DH with PFP techniques. Here, PFP is employed to measure diffuse surface regions, while DH is utilized for specular areas that PFP cannot effectively capture. By capturing both specular and diffuse reflections within the same optical path, the setup aims to achieve high accuracy and efficiency in 3D composite surface measurements.

This study introduces an effective 3D surface reconstruction technique tailored for inspecting IC carrier boards with chips that feature both specular and diffuse surfaces. The remainder of this paper is organized as follows: Section 2 presents the proposed imaging technique for 3D composite surfaces. In Section 3, the experimental setup and results of the 3D composite surface reconstruction for IC and its carrier board inspection are presented and analyzed. Finally, Section 4 summarizes the conclusions of the study.

## 2. Working Principle

### 2.1. Principles and Methods of 3D Composite Surface Imaging

Most real-world objects exhibit both diffuse and specular reflection characteristics, sparking interest in 3D reconstruction techniques for composite surfaces in precision manufacturing processes. To measure surfaces with these complex reflective properties, this paper proposes a technique combining PFP and DH, creating an integrated system to measure the 3D structures of surfaces that have both diffuse and specular reflection.

**Figure 1:**
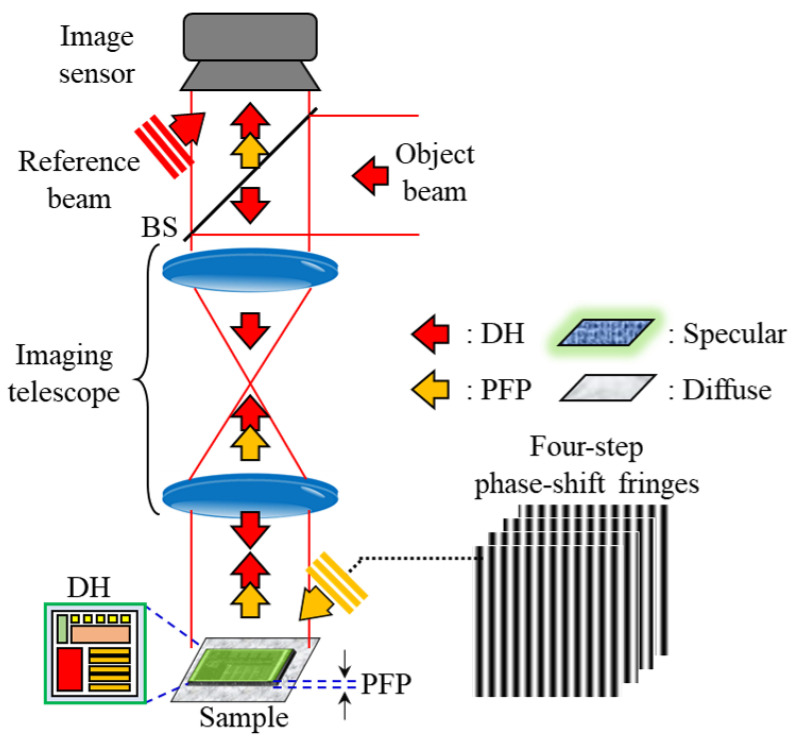
Schematic diagram of 3D composite surface measurement using an integrated setup by engaging DH with PFP optical configuration.

Figure 1 presents the conceptual diagram for 3D composite surface measurement using this integrated system, where the orange arrow represents the optical setup of PFP, and the red represents that of DH. In the PFP subsystem, a four-step phase-shifting fringe projection pattern is employed to measure phase difference information between diffuse and specular surfaces. However, PFP technology faces challenges in accurately resolving the fine details of specular surfaces and in achieving sufficient resolution along the z-axis, limiting the analysis of intricate structural details on specular surfaces.

To address this limitation, we adopted an integrated optical experimental setup combining DH with PFP, enabling the capture of both specular and diffuse reflections along the same optical path. The DH setup specifically measures surfaces with complex specular reflective structures. DH captures the wavefront information of the sample by interference with a reference beam, creating a digital hologram. Through numerical reconstruction, the amplitude and phase of the wavefront can be retrieved from the digital hologram, with the phase information indicating the change in optical path length at each point in the FOV of the sample. This numerical wavefront reconstruction allows us to extract a wrapped phase map (phase information encoded within a specific range), and by analyzing these phase values, we can reconstruct the 3D details of the specular surface’s fine structure.

In this integrated system, PFP is mainly used to rapidly acquire the 3D shape of diffuse surfaces, while DH is used to measure specular surfaces, which PFP cannot easily resolve. Although DH has limitations in analyzing diffuse surface information, it can precisely capture fine structural details on specular surfaces. By combining the measurement results obtained from both PFP and DH, as well as the information from diffuse and specular reflections, we can accurately reconstruct the 3D profile of composite surfaces and the fine structure on specular surfaces. The following subsections provide a detailed explanation of the relevant techniques.

### 2.2. Phase-Shifting Fringe Projection Recording and Reconstruction

**Figure 2:**
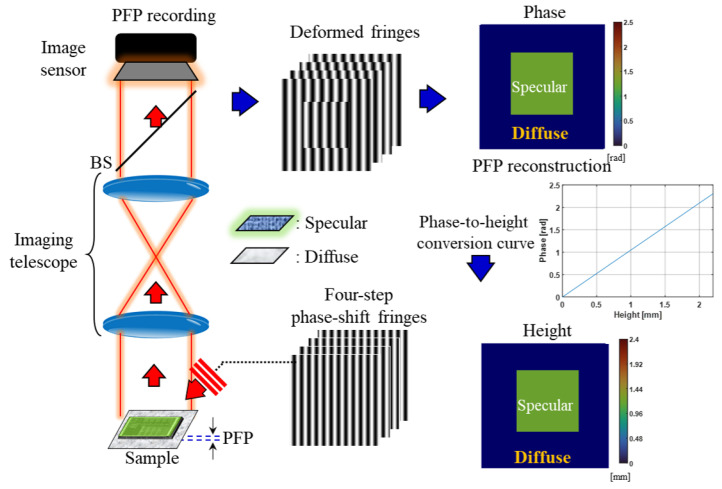
Schematic diagram of the PFP recording and reconstruction.

In the experimental setup of the PFP subsystem, when the fringes are projected onto the sample at an inclined angle, they deform according to the height of the diffuse surfaces. Therefore, by analyzing the deformation of the fringes, the height of the object can be calculated. The flowchart for the PFP recording and reconstruction process is shown in Figure 2. The formula for the four-step phase-shifting fringes, which are projected onto the sample, is based on sinusoidal amplitude grating and can be expressed as $I_{n}=0.5\left[1+\cos\left(2\pi f_{0}x-0.5\left(n-1\right)\pi \right)\right]$, where *n* ranges from 1 to 4, representing the four different phase-shifting fringe patterns, f_0_ is the spatial frequency of the grating, which determines the density of the fringes, and *x* is the spatial coordinate of the sample.

After the fringes are projected onto the sample, the phase information can be retrieved using a phase-shifting reconstruction algorithm for diffuse surfaces. To accurately retrieve the surface phase-to-height information, the fringe analysis of the multi-step phase-shifting algorithms needs to be established for diffuse surfaces, as previously illustrated. In our proposed 3D shape measurement, a reference plane has been set up to obtain the absolute phase map. The phase of the fringe patterns recorded at each measured position encodes the height of the objects. A translation stage is utilized to move the sample, with a movement accuracy of 10 μm in our experiments. The phase information of the measurement plane can be determined relative to the reference plane (z = 0) using phase unwrapping algorithms. This information is then converted into corresponding height using the pre-calibrated phase-to-height conversion. The pre-calibrated phase-to-height conversion curve is derived from the four-step phase-shifting algorithm. In our experiments, their height was approximately 2.2 mm, and the measured reference distance ranged from 0 to 2.2 mm. The absolute phase was demodulated using a linear regression curve with a coefficient of determination (R^2^) of 0.998. The R^2^ measures how well the regression curve fits the data, indicating the proportion of variance in the original data explained by the regression model. R^2^ ranges from 0 to 1, with a value closer to 1 indicating a better fit, meaning the model explains most of the variance. Conversely, an R^2^ value near 0 indicates a poor fit and weak explanatory power. The formula for R^2^ is $1-\sum {\left(y-\hat{y}\right)}^{2}/\sum {\left(y-\bar{y}\right)}^{2}$, where *y* represents the original data, $\hat{y}$ is the predicted value from the regression curve, and $\bar{y}$ is the mean of the original data. Thus, the R^2^ of 0.998 demonstrates a strong relationship between phase and height. The relationship between the phase information and the height of the diffuse surface can be established through fringe analysis and phase retrieval. The phase-to-height conversion curve relating the phase difference to the height map is plotted, as illustrated in Figure 2, providing a representation of this relationship. The accuracy of this measurement process is assessed using the root-mean-square error (RMSE), which is found to be 1.96% for measured distances within 2.2 mm, with a standard error of 0.016 mm. The PFP subsystem is established to achieve a precise height measurement of composite surfaces by analyzing the deformation of projected fringes. The combination of mathematical modeling, calibration of fringe analysis, and accurate measurement techniques enhance the reliability of the experimental setup, facilitating effective 3D shape reconstruction of diffuse surfaces in various applications.

### 2.3. Digital Holography Recording and Reconstruction

**Figure 3:**
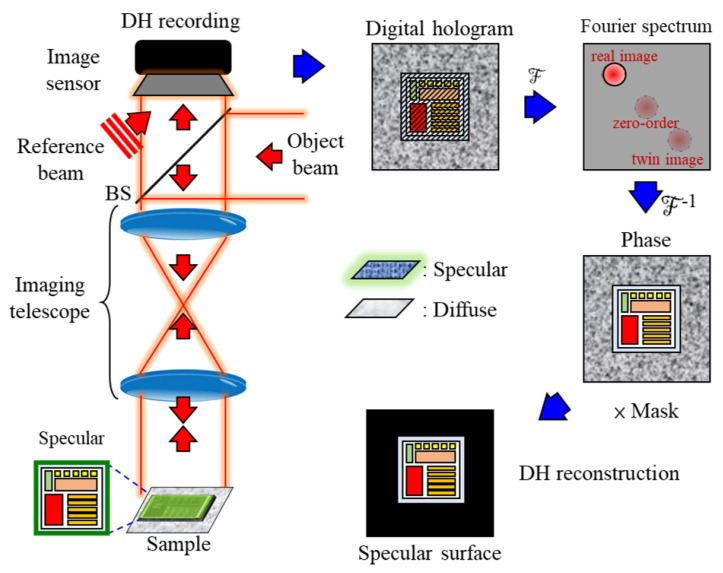
Schematic diagram of the DH recording and reconstruction.

Due to the composite characteristics of object surfaces, developing an algorithm to distinguish between specular and diffuse reflection is crucial for the 3D reconstruction of composite surfaces. DH is primarily utilized to reconstruct the 3D shape of specular surfaces. The flowchart for the DH recording and reconstruction process is shown in Figure 3.

In this method, information about the specular surface is captured in digital holograms, allowing for the detection and reconstruction of the 3D shape of the specular surface from the acquired hologram. Once the detection procedure for the specular surface is analyzed, the shape can be reconstructed through the following steps.

First, the wavefront information of the sample, denoted as O(x,y), is recorded to form a hologram through interference with a reference beam R(x,y). The recorded hologram can be expressed as $H\left(x,y\right)={\left|O\left(x,y\right)+R\left(x,y\right)\right|}^{2}$. Upon expanding this formula, it can be rewritten as ${\left|O\left(x,y\right)\right|}^{2}+{\left|R\left(x,y\right)\right|}^{2}+O\left(x,y\right)R^{*}\left(x,y\right)+O^{*}\left(x,y\right)R\left(x,y\right)$, where the symbol ∗ denotes the conjugate term. Each interference term can be separated using the Fourier transform algorithm, where ${\left|O\left(x,y\right)\right|}^{2}+{\left|R\left(x,y\right)\right|}^{2}$ represents the zero-order term, while $O\left(x,y\right)R^{*}\left(x,y\right)$ and $O^{*}\left(x,y\right)R\left(x,y\right)$ correspond to the real image and twin image, respectively.

To reconstruct the wavefront information of the sample, a mask is generated to select the real image region $O\left(x,y\right)R^{*}\left(x,y\right)$ in the Fourier domain. The selected region undergoes inverse Fourier transform and is multiplied by the numerically reconstructed wave R(x,y) to obtain the complex wavefront information of the sample.

However, in the DH reconstruction results, interference terms do not form in diffuse regions, leading to randomly distributed phase information in those areas. To address this, we design masks to filter out random signals, enhancing the visibility of fine structures on the specular surface.

### 2.4. Three-Dimensional Composite Surface Reconstruction

**Figure 4:**
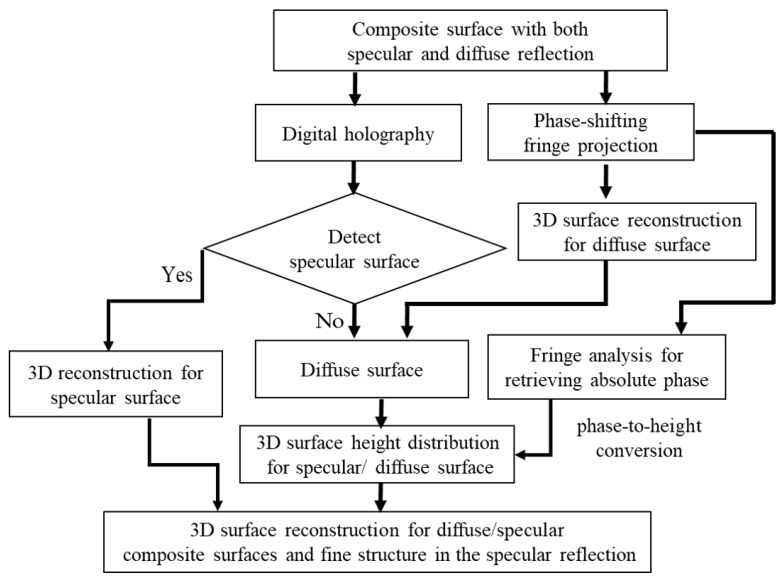
Flowchart of 3D composite surface reconstruction in an integrated system.

In this study, we propose a 3D composite surface reconstruction method based on an integrated system, and the flowchart is shown in Figure 4. To measure the 3D shape of composite materials, the integrated system engages DH with PFP for specular and diffuse reflected surfaces measurements, respectively.

The DH technique is used to record digital holograms that contain wavefront information of the sample. Interference patterns from DH are only observable on specular surfaces, not on diffuse reflective ones. Consequently, the clarity of the interferogram and phase images is employed to determine whether each position on the sample corresponds to specular or diffuse surfaces. If the interferogram is clearly visible, the DH configuration is utilized to reconstruct and reveal a more precise distribution of the fine structure of the specular surfaces. Conversely, if no interferogram is observed, the reconstructed phase in that area will exhibit a random distribution, making it challenging to accurately discern the sample structure.

The PFP technique captures four-step phase-shifting deformed fringe images of the objects, which allows us to perform fringe analysis and extract absolute phase information. This absolute phase can subsequently be converted into height information through a phase-to-height conversion process.

After reconstructing the samples using PFP and DH, respectively, the height information obtained from the PFP was integrated into the overall 3D reconstruction. The average height of the specular surface was derived from the average height calculated using the phase information produced by the PFP technology, while the fine structure distribution relied on the results from the DH subsystem. In the diffuse regions of the test sample, the reconstructed phase from DH did not yield a clear representation of the sample structure, mirroring the results obtained from PFP.

In the proposed integrated experimental setup, both specular and diffuse reflected light are effectively captured along the same optical path, enabling simultaneous full-field surface reconstruction of the 3D shape of the composite surface.

## 3. Experimental Setup and Results

**Figure 5:**
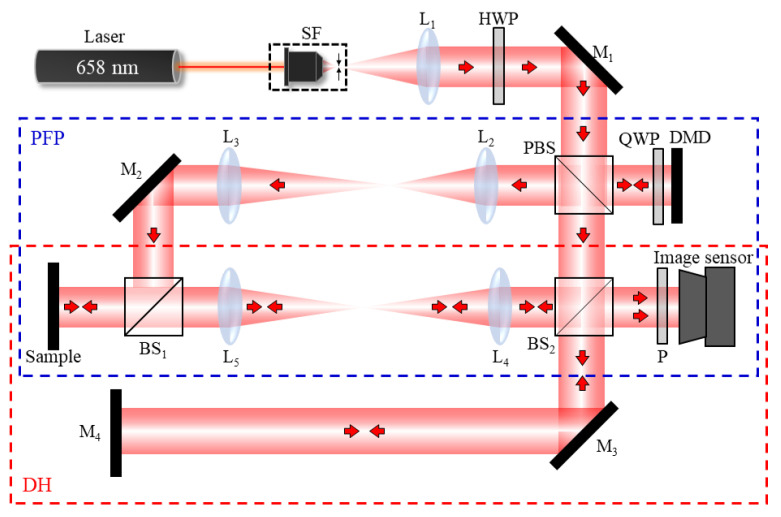
Integrated system setup for 3D composite surface measurement. (Blue dashed line represents the PFP part, and the red dashed line represents DH optical configurations. SF: spatial filter, L: lens, HWP: half-wave plate, M: mirror, PBS: polarizing beam splitter, QWP: quarter-wave plate, DMD: digital micromirror device, BS: beam splitter, P: polarizer.)

The experimental setup of the integrated optical configuration by engaging DH with PFP is shown in Figure 5. The integrated system employs a laser light source with a wavelength of 658 nm (Raise, Taiwan). The laser beam is expanded using a beam expander (SF+L_1_) and subsequently passes through a half-wave plate (HWP) and a polarizing beam splitter (PBS) to split the beam into two subsystems. The blue dashed line indicates the PFP subsystem, which is positioned in the reflection arm of the PBS (s-wave), while the red dashed line denotes the DH subsystem, located in the transmission arm of the PBS (p-wave).

To accommodate samples with composite surfaces, the polarization state can be adjusted with the HWP, allowing measurements to be taken using either the PFP subsystem (for diffuse surfaces) or the DH subsystem (for specular surfaces). In the PFP subsystem, a digital micromirror device (DMD, Texas Instruments, US; panel: DLM471TEEVM; control board: DLPC7540EVM; pixel number: 1920 × 1080; pixel size: 5.4 μm) is employed to project the fringe pattern, which is designed with a fringe period of 172.8 μm. To address the polarization characteristics of the PBS (s-wave), a quarter-wave plate (QWP) is positioned between the PBS and the DMD to modify the polarization state. As the beam reflects along the optical path, its polarization state transitions from s-wave to p-wave, enabling the wavefront containing the fringe information to pass through the PBS. Subsequently, the fringe wavefront is directed through an imaging system composed of lenses L_2_ (f = 200 mm) and L_3_ (f = 250 mm) to form an image of the fringes on the sample. Since PFP requires the inclined fringe pattern to be incident on the sample, the angle of the mirror (M_2_) is adjusted to tilt the fringe pattern towards the sample. Following this, a second imaging system consisting of lenses L_4_ (f = 200 mm) and L_5_ (f = 200 mm) captures the object and the distorted fringe information onto an image sensor (Pixoel, Taiwan; model: UI-1540SE-M-GL; pixel number: 1280 × 1024; pixel size: 5.2 μm). Phase shifting is achieved by altering the projected fringes on the DMD, which records multiple deformed images.

The DH subsystem is constructed based on a modified Michelson interferometer. The beam is split into an object beam and a reference beam using a beam splitter (BS_2_). The wavefront information of the sample is collected through a second imaging stage, where it interferes with the reference beam to form a digital hologram that is recorded by the image sensor. Since both the PFP and DH subsystems share the same imaging system and image sensor, they maintain consistent system resolution (7.81 μm) and fields of view (FOV: 6.66 × 5.32 mm^2^), facilitating image processing for composite surfaces.

**Figure 6:**
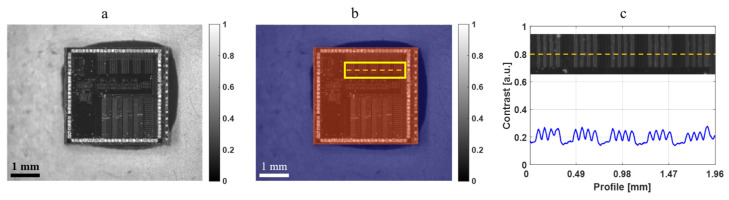
Measured IC chip. (**a**) Image of bright-field microscopy. (**b**) Digital staining: the caramel-colored region represents the IC chip region, and the purple region represents the carrier board region. (**c**) Striped structures in the yellow frame of (**b**).

To verify the 3D morphology measurement of composite surfaces using the optical configuration that integrates DH and PFP, an IC chip is used as an example. Figure 6a displays a bright-field microscopy image of the IC chip, which can be divided into caramel-colored and purple regions, as illustrated in Figure 6b. The caramel-colored region represents the chip, which measures approximately 2.86 × 2.72 mm^2^, while the purple region corresponds to the carrier board. The height difference between the chip and the carrier board is about 0.74 mm, as measured by a vernier caliper. Additionally, striped structures are visible in the upper right corner of the chip, as shown in Figure 6c, where each stripe has a width of approximately 66 μm. These striped structures in the yellow frame will serve as features for subsequent investigations into the composite surface measurements.

**Figure 7:**
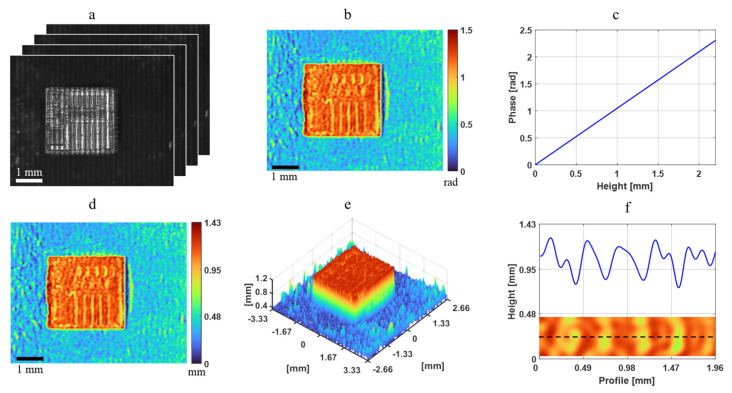
Experimental results of PFP. (**a**) Recorded deformed fringe patterns. (**b**) Reconstructed phase image. (**c**) Phase-to-height conversion. (**d**) Phase distribution. (**e**) Three-dimensional view of the height distribution for IC carrier board and chip. (**f**) Profile of IC chip with fine structure.

The PFP imaging results of the IC chip are presented in Figure 7. Figure 7a displays the recorded images of the deformed four-step phase-shifting fringe patterns. Using the four-step phase-shifting reconstruction method, the PFP phase results can be reconstructed, as shown in Figure 7b. The average phase difference between the chip region and the carrier board region is approximately 0.776 rad. By applying the phase-to-height conversion curve (Figure 7c), the sample phase reconstructed by PFP can be converted into height measurements, as illustrated in Figure 7d. The resulting height distribution provides a 3D view of the IC chip, as shown in Figure 7e. After conversion, the height difference between the chip region and the carrier board region is approximately 0.74 mm. Additionally, due to the specular nature of the chip region, resolving the structural distribution from the PFP reconstructed image is challenging. Consequently, when plotting the profile at the same position as observed in the bright-field microscope (Figure 7f), it is not possible to accurately analyze the structural distribution from the profile.

**Figure 8:**
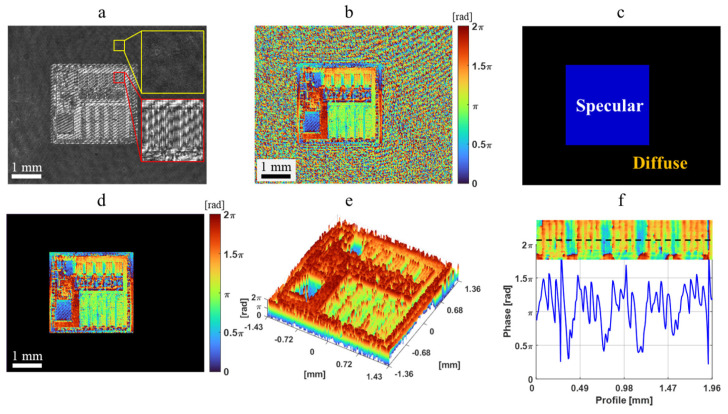
Experimental results of DH. (**a**) Recorded hologram. (**b**) Reconstructed phase image. (**c**) Mask generation for the composite region defined by interferogram and phase distribution. (**d**) Mask design. (**e**) Three-dimensional view of the IC chip. (**f**) Profile of IC chip with fine structure.

The DH imaging results of the IC chip are presented in Figure 8. Figure 8a displays the hologram recorded by DH, where a distinct interferogram is observable in the chip region, while no fringes appear in the carrier board region. After applying the angular spectrum algorithm for DH numerical reconstruction, the phase image of the sample was successfully reconstructed, as shown in Figure 8b. This phase reconstruction image reveals the structural distribution of the sample within the chip region. In the carrier board region, the diffuse surface nature results in the absence of the interferogram, leading to a random phase distribution.

The specular and diffuse regions can be differentiated based on the interferogram and phase images, where the blue region indicates the specular surface, and the black region denotes the diffuse surface. To minimize the impact of the diffuse surface, a mask is generated (Figure 8c) to filter out the diffuse region, thus highlighting the chip structure, as illustrated in Figure 8d. The 3D view of the chip region is displayed in Figure 8e.

Subsequently, a profile of the structure is plotted at the same position as observed by the bright-field microscope, as shown in Figure 8f. The measured width by DH is approximately 64 μm, with a deviation of about 3% from the results obtained from the bright-field microscope. These results demonstrate that DH exhibits powerful imaging capabilities on specular surfaces. Additionally, variations in the phase value reflect changes in the height of the chip.

**Figure 9:**
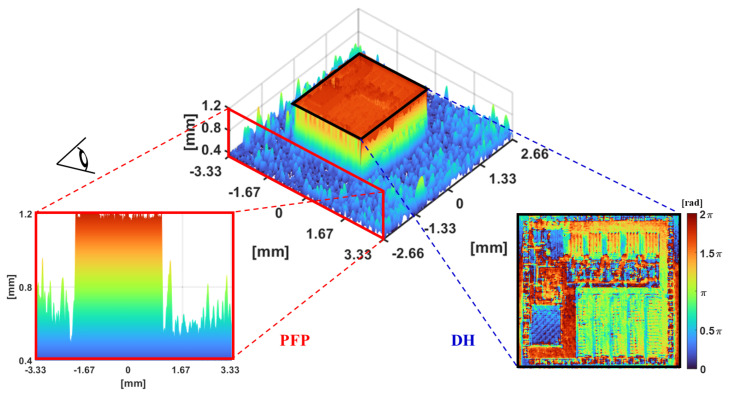
Three-dimensional surface reconstruction for specular/diffuse composite surfaces.

The IC carrier board with a chip, which exhibits both specular and diffuse reflections on its surface, was measured. The 3D surface reconstruction results obtained from the proposed integrated system are shown in Figure 9. The detection of the specular surface is determined through interferogram analysis of the digital hologram, enabling the identification of regions with specular and diffuse surfaces. After projecting the fringes onto the IC carrier board, the phase information for the diffuse surface can be retrieved using the phase-shifting reconstruction algorithm, as indicated by the red dashed line in Figure 9.

To extract surface phase-to-height information, a multi-step phase-shifting fringe analysis is conducted for the diffuse surface within the red dashed line in Figure 9. In contrast, the 3D surface reconstruction represented by the blue dashed line corresponds to the results for the specular surface of the IC chip. By utilizing an integrated experimental setup that combines DH and PFP configurations, we successfully captured specular light, allowing for the detailed reconstruction of the fine structure on the specular surface. The experimental results demonstrate that the proposed technique effectively detects and accurately reconstructs the 3D shape of the inspected IC carrier board, which features both specular and diffuse composite surfaces.

## 4. Conclusions

In summary, we have proposed and experimentally demonstrated an effective 3D shape metrology technique that integrates digital holography into the fringe projection setup for measuring composite surfaces. Fringe projection is employed to retrieve phase information from 3D diffuse surfaces, while the digital holography subsystem effectively reconstructs holograms of specular surfaces that cannot be measured effectively using fringe projection alone. This integrated optical configuration enables the simultaneous acquisition of both specular and diffuse reflected light within a single experimental setup and the same FOV using a single-wavelength technique. This streamlined design simplifies the setup, eliminates alignment challenges associated with separate systems, and enhances the efficiency of full-field surface profilometry. However, while DH has proven effective in reconstructing specular regions, accurately measuring the stepped fine-strip structures using single-wavelength DH remains challenging. To address this limitation, future studies could explore dual-wavelength techniques or other advanced methods for achieving continuous phase unwrapping. These approaches could improve measurement accuracy and enhance the system’s applicability to practical scenarios. Furthermore, to enable depth measurements for composite surfaces, the proposed technique integrates fringe analysis with phase unwrapping algorithms, thereby facilitating phase-to-height conversion. The experimental results of the proposed system in measuring complex surfaces, such as integrated circuits and their carrier boards, highlight its potential for widespread application in fields requiring detailed and precise 3D surface measurements.

## Data Availability

The dataset underlying the results presented in this paper is not publicly available at this time but may be obtained from the authors upon reasonable request.
